# Nature, Source and Function of Pigments in Tardigrades: *In Vivo* Raman Imaging of Carotenoids in *Echiniscus blumi*

**DOI:** 10.1371/journal.pone.0050162

**Published:** 2012-11-21

**Authors:** Alois Bonifacio, Roberto Guidetti, Tiziana Altiero, Valter Sergo, Lorena Rebecchi

**Affiliations:** 1 Centre of Excellence for Nanostructured Materials and Department of Engineering and Architecture, University of Trieste, Trieste, Italy; 2 Department of Life Sciences, University of Modena and Reggio Emilia, Modena, Italy; 3 Department of Education and Human Sciences, University of Modena and Reggio Emilia, Reggio Emilia, Italy; The Australian National University, Australia

## Abstract

Tardigrades are microscopic aquatic animals with remarkable abilities to withstand harsh physical conditions such as dehydration or exposure to harmful highly energetic radiation. The mechanisms responsible for such robustness are presently little known, but protection against oxidative stresses is thought to play a role. Despite the fact that many tardigrade species are variously pigmented, scarce information is available about this characteristic. By applying Raman micro-spectroscopy on living specimens, pigments in the tardigrade *Echiniscus blumi* are identified as carotenoids, and their distribution within the animal body is visualized. The dietary origin of these pigments is demonstrated, as well as their presence in the eggs and in eye-spots of these animals, together with their absence in the outer layer of the animal (i.e., cuticle and epidermis). Using *in-vivo* semi-quantitative Raman micro-spectroscopy, a decrease in carotenoid content is detected after inducing oxidative stress, demonstrating that this approach can be used for studying the role of carotenoids in oxidative stress-related processes in tardigrades. This approach could be thus used in further investigations to test several hypotheses concerning the function of these carotenoids in tardigrades as photo-protective pigments against ionizing radiations or as antioxidants defending these organisms against the oxidative stress occurring during desiccation processes.

## Introduction

Tardigrades, or water bears, are microscopic aquatic animals (100–1000 µm in length), most of which belong to the group of desiccation-tolerant multicellular organisms [1], [2]. Although tardigrades colonized a wide range of ecosystems and habitats all over the world, the highest number of species is found in terrestrial ecosystems where they often constitute the major component of the fauna in lichens and mosses. These habitats provide a “sponge-like” environment featuring a myriad of small pockets of water, in which tardigrades live. Such habitats, however, are subject to occasional periods of desiccation or freezing: as mosses and lichens lose water, tardigrades desiccate or freeze consequently, entering in physiological states called “anhydrobiosis” and “cryobiosis”, respectively. In such states, grouped under the general name of “cryptobiosis”, metabolic activity is suspended because of absence of liquid water, until environmental conditions return favourable [1], [2]. Tardigrades can persist in the anhydrobiotic state even for years, during which they are able to withstand several physical and chemical extremes [1]–[5], including exposure to vacuum and both solar and cosmic ionizing radiation as found in low Earth orbit space [3], [4], [6], [7]. Remarkably, even in their hydrated active state, tardigrades show an unexpectedly high survival rate upon freezing (even with temperatures as low as −196°C) [8], [9] or exposure to high levels of ionizing radiations [3], [5]. The biochemical and physiological mechanisms underlying the extraordinary resistance of both desiccated and hydrated tardigrades to such harsh conditions are currently little known and constitute an intriguing challenge for biologists and biochemists.

Regardless of the wide range of colonised environments, tardigrades do not exhibit a great variation in internal organ structure and organization. Their body, invariably constituted by an anterior region (or head region) followed by four trunk segments with a pair of legs each, is covered by a chitinous cuticle that must be shed as the organism grows and by a monolayer of flat epidermal cells [10]. All tardigrades have muscular, nervous, reproductive and digestive systems, and their body cavity, which is rich in free-floating storage cells, allows efficient nutrition and gas exchange with no need for circulatory or respiratory systems [10]. However, in spite of this uniformity in body structure, tardigrades exhibit a variety of pigmentations: several tardigrade species are translucent or whitish, but many other are colored, sometimes markedly, with a variety of shades of red-orange, brown, green and yellow pigments [10], [11]. These pigments can be uniformly or unevenly distributed in the body cavity, or may be located in storage cells or in the epidermis cells. Sometimes, the animal pigmentation may be caused by gut content [10], [11]. Moreover, dark or red pigmented eye-spots may be present on the head region in close association with the outer dorso-lateral lobe of the brain, depending on the genus or species [10], [12]. Each eye-spot consists of a single pigment cup cell, filled with spherical electron-dense pigmented granules, one or two microvillous sensory cells, and ciliary sensory cells [12].

Within tardigrades, the genus *Echiniscus* is the most rich in the number of species, and most of them are brightly coloured [13]. Despite the occurrence of pigments is well documented for many *Echiniscus* species and many other genera belonging to the family Echiniscidae, their chemical nature as well as their function remains unknown, especially in relation to their resistance to harsh physical and chemical conditions. Some hypotheses were formulated about these pigments [14], [15], but to the best of our knowledge no direct and conclusive experimental proof about their structure, source or function has been reported. In order to resolve these open questions, approaches involving techniques capable of performing *in-vivo* chemical analysis on single tardigrades are highly desirable. The low sample quantity available and the *in-vivo* requirement rule out most of the traditional analytical methods such as separation-based, electrochemical or mass spectrometry techniques. Raman micro-spectroscopy, however, has all the characteristics required for such a task.

Raman spectroscopy is a non-destructive, semi-quantitative analytical technique based on the scattering of laser radiation by vibrating molecules that has proved to be an ideal tool for studying living cells and biological tissues [16], [17]. Information about the biochemical species present in a sample can be simply obtained by illuminating it with a low-power laser and then analysing the scattered light. Since it originates from molecular vibrations, a Raman spectrum closely depends on molecular structure and is thus unique to each molecule, in principle allowing the identification of chemical species on a spectral basis [18]. The level of structural detail conveyed by Raman spectroscopy ranges from the identification of the complete molecular structure to a general classification of the molecular class of the analyte of interest, depending on its spectral characteristics as well as on the chemical complexity of the sample. Moreover, the intensity of the Raman scattering can be exploited as a “contrast mechanism” to image the spatial distribution of the main biochemical constituents of a biological sample (i.e. Raman mapping or imaging).

In this paper we unambiguously identify the pigments in the heterotardigrade *Echiniscus blumi* Richters 1903 as carotenoids, demonstrate their food origin and set-up a method to study their anti-oxidant function in these animals by *in-vivo* spectroscopic and imaging techniques based on Raman scattering. Using this semi-quantitative methodology based on Raman imaging, changes in carotenoid content are monitored in living tardigrades undergoing oxidative stress.

## Materials and Methods

### Tardigrade Collection

Animals and exuvia with eggs of the moss-dwelling and anhydrobiotic heterotardigrade *Echiniscus blumi* Richters 1903 were extracted from the moss *Grimmia orbicularis* Bruch ex Wilson growing on a rocky outcrop at Sasso Tignoso (Modena, Italy; N44°12.837′, E10°33.237′, 1382 m a.s.l.). In order to extract tardigrades, moss was maintained in water for about 30 min. After soaking, specimens were separated using sieves, collected under a stereomicroscope and maintained over 4 h in water (natural mineral water/distilled water; 1∶1) at 20°C before any experiments. No specific permits were required for the described field studies, which did not involve endangered or protected species.

### Raman Spectroscopy and Imaging Measurements

**Figure 1:**
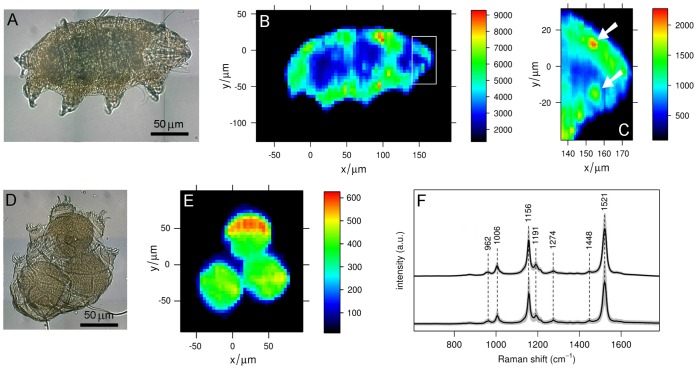
Raman maps of carotenoid pigments in *E. blumi*. (A) Bright field micrograph and (B,C) intensity Raman maps of a living *E. blumi*; Raman map C covers the anterior region of the tardigrade (white rectangle in map B). (D) Bright field micrograph and (E) intensity Raman map of an exuvium containing three eggs of *E. blumi*. (F) Average Raman spectra (black) and intensity standard deviation (grey) of Raman maps B and E; spectral intensity was re-scaled for better comparison. All Raman maps depict the relative concentration of carotenoid species, measured as the integrated Raman intensity between 1501 cm^−1^ and 1541 cm^−1^, corresponding to the intense ν_1_ C = C stretching vibration band of carotenoids. In C, white arrows indicate spots with high carotenoid concentration corresponding to the eye-spots. The color scale bar on the right of each Raman map has units of total photon counts/Δcm^−1^ for the Raman shift interval considered.

**Figure 2:**
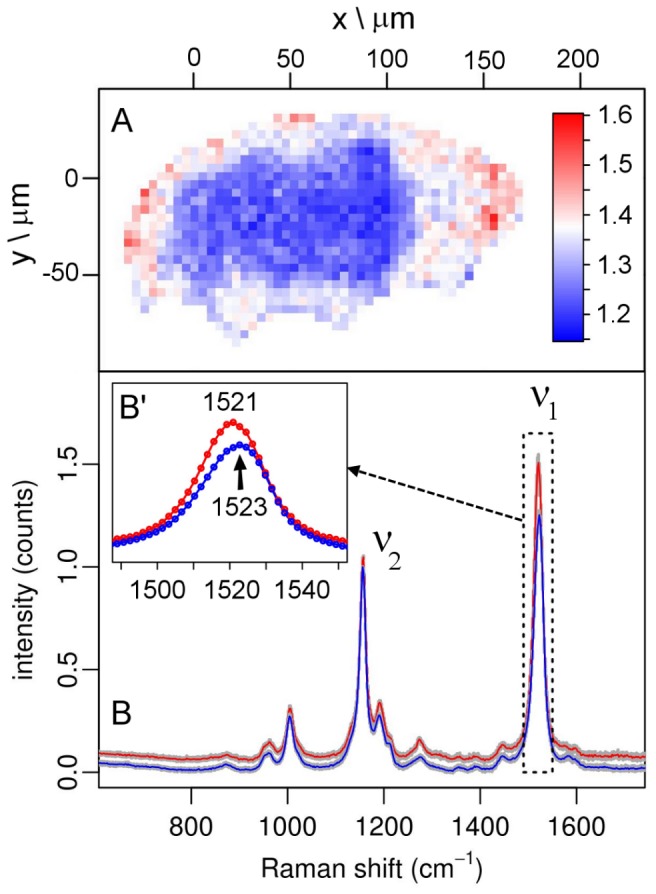
Variety of spectral characteristics for carotenoids within a *E. blumi* specimen. (A) Image depicting the ν_1_/ν_2_ band intensity ratio (“IR”, color scale bar on the right) for the Raman map shown in Fig. 2B. (B) Average normalized intensity of the two subsets of spectra having the lowest and the highest ν_1_/ν_2_ ratio, in blue (IR<1.2, 26 spectra) and red (IR>1.45, 21 spectra), respectively; for each subset, the intensity of standard deviation is reported in grey. (B’) Inset with detail of ν_1_ bands.

**Figure 3:**
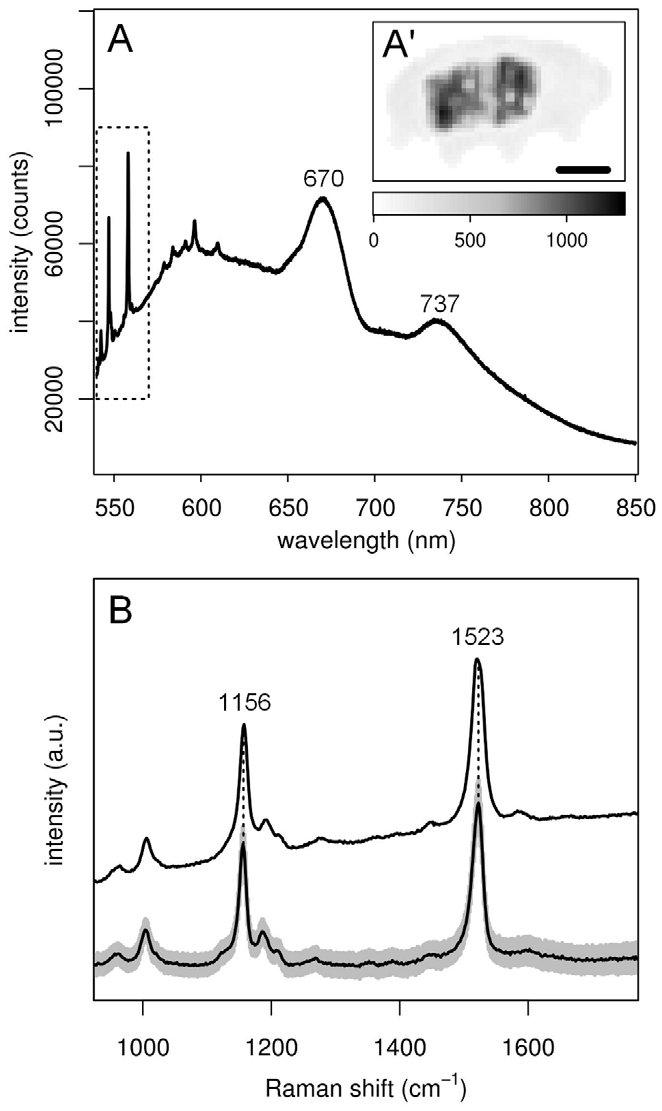
Food origin of carotenoids. (A) Photoluminescence emission spectrum from the gut of a living *E. blumi* (excitation at 514.5 nm). Raman bands due to carotenoids (dotted box) are observed together with the chlorophyll fluorescence emission bands at 670 and 737 nm; (A’) inset with fluorescence intensity map (emission at 670 nm); black bar is 50 µm, grey scale bar on the bottom of the Raman map has units of counts at 670 nm. (B) Bottom spectrum: average normalized spectrum (black) and intensity standard deviation (grey) of a set of 300 Raman spectra collected from the moss leaves of *G. orbicularis*; top spectrum: Raman spectrum from tardigrade’s gut (dotted box in A).

**Figure 4:**
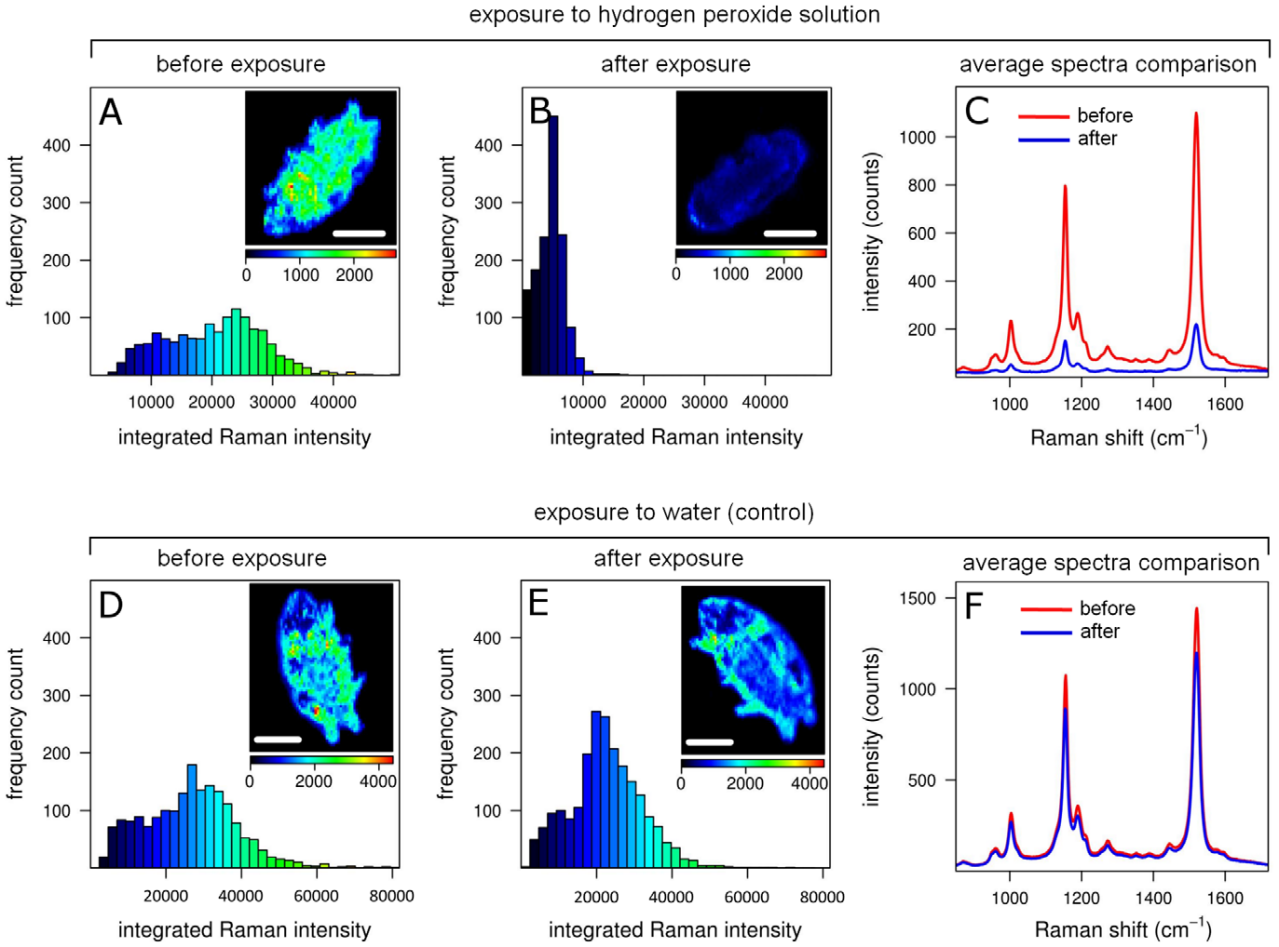
Effect of induced oxidative stress on the carotenoid content. (A–B, D–E) Histograms of the integrated Raman intensity in the 1460–1570 cm^−1^ region from Raman maps of two living *E. blumi* specimens before any treatment (A, D) and after exposure to 25 mM hydrogen peroxide (B, treated) or water (E, control) for 15 min. For each histogram, the corresponding intensity Raman map depicting the carotenoids distribution (i.e. the intensity at 1521 cm^−1^) is shown as inset. White scale bars = 200 µm, color scale bars have units of counts. (C, F) Average spectra of the Raman maps before (red) and after (blue) exposure to hydrogen peroxide (maps in A, B) or water (maps in C, D).

All Raman and photoluminescence spectra and maps were acquired using an InVia micro-Raman spectrometer (Renishaw plc, Wotton-under-Edge, UK) equipped an argon ion laser emitting 50 mW at 514.5 nm (Modu-Laser, Centerville, UT) and a motorized stage (Prior, Cambridge, UK). The laser was focused on samples via a 60×objective (N.A. 1.00), its power being below 1 mW at the sample and kept constant during each measurement. Raman maps were collected in the fast-imaging Streamline™ mode, with acquisition times varying from approximately 3 to 5 min (depending on the map size), and with lateral resolutions (i.e. step sizes) of 1.1 µm (Fig. 1C), 4.3 µm (Figs. 1B, 1E, 2A, and 3A’) or 5.4 µm (Fig. 4 and Figs S3 ad S4 in SI). Data pre-processing (cosmic rays removal, baseline correction, normalization) and analysis, as well as image production, was performed with hyperSpec package (http://hyperspec.r-forge.r-project.org) for R [19].

For Raman measurements, tardigrades were singularly put in a drop of water between a CaF_2_ slide (Crystal GmbH, Berlin, Germany) and a 15×15 mm, 0.5 mm thick glass coverslip, in such a way that the animals were slightly compressed and unable to move. After collection of Raman data, each specimen was put again in water to check its viability on the basis of active movements of its body. All the data reported were collected from tardigrades which satisfied the viability criterion before and after the Raman measurements.

### Test to Evaluate the Effects of H_2_O_2_ on Tardigrade Survival

Ten different final concentration of H_2_O_2_ (5, 10, 20, 25, 35, 45, 55, 65, 75 and 150 mM) were tested. For each H_2_O_2_ concentration, five replicates each of 5 hydrated animals were immersed for 15 min in 300 µl of H_2_O_2_ (30% w/w) in water at 20°C. Then, animals were transferred in water and their viability was verified after 1 h and 24 h from the end of the stress. As control, 5 replicates each with 5 tardigrades were maintained in water for 15 min at 20°C. The survival of *E. blumi* treated with H_2_O_2_ is shown in SI (Fig. S2). The percentage survival of control tardigrades was 100%.

### Measurements of Carotenoids Content in Tardigrade before and after H_2_O_2_ Treatment

Oxidative stress was individually induced in 8 *E. blumi* specimens (i.e. 8 independent replicates: experimental group) upon exposure to 300 µl of H_2_O_2_ 25 mM for 15 min at 20°C. As control group, 8 specimens individually (i.e. 8 independent replicates) maintained in water for 15 min at 20°C were used. For each specimen in the experimental group, two Raman maps were collected, one before H_2_O_2_ exposure and the other one 1 h after the end of H_2_O_2_ exposure. For the control group, the two Raman maps were collected immediately before and 1 h after immersion in water. For both the experimental and control groups, the tardigrade viability was checked after the former Raman map collection, after H_2_O_2_ exposure, after the latter Raman map collection, and 24 h after the end of treatment.

A first estimate of the total carotenoid content in *E. blumi* specimens was made upon comparing the intensity of the average Raman spectrum of the map of each animal before and after H_2_O_2_ exposure. Spectra associated to background pixels were not taken into account for averaging. For a more accurate estimate, histograms were calculated and compared, showing the distribution of the integrated Raman intensity calculated for the ν_1_ band (1460–1570 cm^−1^). The *total integrated Raman intensity* for the same band was calculated by summing the integrated intensities from all the spectra in a map, and it was taken as a third estimate of the total carotenoid content.

## Results

### Carotenoid Nature and Localization of the Pigments

The typical average Raman spectrum of living *E. blumi* specimens (Fig. 1) is completely dominated by characteristic carotenoid bands which are observed in spectra excited with a blue or green laser, such as the intense ν_1_ band at 1521 cm^−1^ arising from stretching vibrations of conjugated C = C bonds [20]. Bands due to other usual biochemical species such as proteins, sugars, lipids and nucleic acids are not observed because their Raman signal is overwhelmed by carotenoid bands, whose intensity is selectively enhanced by several orders of magnitude via the resonance Raman (RR) effect [20], [21].

A Raman shift of 1521 cm^−1^ for the ν_1_ band, the relatively weak ν_4_ band at 962 cm^−1^ and the intensity pattern for the ν_2_ bands cluster (between 1150 and 1200 cm^−1^) suggest an average molecular structure for carotenoids consisting of an undistorted, linear main carbon chain having 11 conjugated C = C double bonds in an “all-trans” configuration [20], [22], [23].

A qualitative picture of the spatial distribution of pigments within *E. blumi* specimens can be visualized upon mapping the Raman intensity of the most intense carotenoid band (Fig. 1). Carotenoids are present inside the whole body cavity, but they appear to be absent in the outer layer of the animal (i.e. cuticle and epidermis) as well as in the claws and in the buccal-pharyngeal apparatus (which is located within the head region). In the tardigrade investigated in Fig. 1, the carotenoid concentration in the body cavity is higher than in the region corresponding to the gut (Fig. 1B), which appears as a dark pigmented area in the middle of the animal body (Fig. 1A). Not all the specimens investigated present such differences in carotenoid concentration between the gut and the body cavity (see Figs. S3 and S4 in SI). On the other hand, all the tardigrades examined in this study show an overall decrease of carotenoid concentration in the head region, with the exception of two small round areas corresponding to the tardigrade eye-spots, where the carotenoid concentration is higher (Fig. 1C). The same carotenoids observed in the body cavity appear to be present, although with a more homogeneous distribution, in the yolk of *E. blumi* eggs (Fig. 1E), whose average Raman spectrum (Fig. 1F, bottom) is identical to that of the animal (Fig. 1F, top). Conversely, the exuvium (i.e. the cuticle left by tardigrades during moulting and used as container for the laid eggs) does not show carotenoid Raman bands, further indicating that the cuticular structures of this species lack such pigments.

Upon a more detailed analysis, slight differences in band position and relative intensities appear between spectra from different parts of the tardigrade body (Fig. 2). Specifically, the ratio between the intensities of the ν_1_ and the ν_2_ bands in the spectra of carotenoids localized in the body cavity and in the eye-spots is higher than that of carotenoids in the gut (Fig. 2A, B; pixels in red correspond to higher intensity ratio), indicating a variation in the relative composition of the molecular structure between the pigments located in those different parts of the tardigrade. As a further indication of such differences, there is an inverse correlation between the ν_1_/ν_2_ intensity ratio and the position of the ν_1_ band (Fig. 2B’), so that in spectra with a higher ratio the ν_1_ band occurs at a slightly lower Raman shift, with a maximum difference of 2 cm^−1^. Such differences in Raman shift of the ν_1_ band for carotenoids localized in the gut of tardigrade suggest that they have a lower number of conjugated C = C bonds with respect to the carotenoids observed in the body cavity.

### Dietary Origin of the Pigments

Raman spectra collected from leaves of the moss *G. orbiculari*s, which constitutes the habitat of *E. blumi* population considered in this paper, are identical to those acquired from the animal’s gut (Fig. 3), indicating that the carotenoids observed in the tardigrade are the same as those present in the moss, thus revealing their dietary origin. As inferred from the average Raman intensities, carotenoids are much (i.e. approximately 20 times) less concentrated in moss leaves than in the tardigrade (Fig. S1). The presence of moss cellular material in the digestive system of this tardigrade is further confirmed by the occurrence of characteristic chlorophyll fluorescence bands in the photoluminescence emission spectra (Fig. 3A) collected from the gut of the same specimen investigated in Figs. 1 and 2. Upon mapping fluorescence intensity at 670 nm, chlorophyll appears to be specifically found in the tardigrade gut (Fig. 3A’), co-localized with those carotenoids having a lower ν_1_/ν_2_ intensity ratio and a ν_1_ band at 1523 cm^−1^ (blue area in Fig. 2A), which are the same spectral features of moss carotenoids (Fig. 3B).

### Semi-quantification of Oxidative Stress-related Effects on the Pigments

Experiments on *E. blumi* specimens showed that the percentage survival, at both 1 h and 24 h after inducing the oxidative stress, sharply decreased upon exposure to H_2_O_2_ concentrations higher than 65 mM, reaching 0% at 150 mM (see Fig. S2 in SI).

According to these data we decided to induce oxidative stress upon exposing tardigrades to 25 mM H_2_O_2_ (i.e. a concentration permitting high stress compatible with high survival) and then, by exploiting the correlation between the concentration of carotenoids and the intensity of their Raman signal [18], [24], [25], to perform a semi-quantitative analysis of these pigments in living tardigrades before and after H_2_O_2_ exposure.

In tardigrades exposed to induced oxidative stress, carotenoid Raman bands clearly decrease in intensity (Fig. 4A–C) with respect to the control group (Fig. 4D–F), indicating a decrease in the amount of carotenoids. This decrease in the carotenoid content upon H_2_O_2_ exposure has been observed for all the 8 specimens of the experimental group (Figs. S3 and S6 in SI), all of which passed the viability criteria after the oxidative stress. Conversely, the 8 specimens of the control group, all of which passed the viability criteria after the collection of Raman maps, did not show any significant decrease in Raman intensity of the carotenoid bands (Figs. S4 and S6 in SI).

It should be noted that un-treated specimens, in both experimental and control groups, showed remarkable inter-individual differences in total carotenoid content, as inferred from the total integrated Raman intensity.

## Discussion

Massonneau and May hypothesized the carotenoid nature of the pigments in *Echiniscus* tardigrades on the basis of solubility tests and chemical reactions [15]. However, since their work in 1950, no other experimental proof has been reported to corroborate their proposition, which remained a well-grounded hypothesis. The Raman spectra reported in this paper definitely decide this issue, unambiguously identifying these pigments as carotenoids having on average a main carbon chain with 11 conjugated C = C bonds in an “all-trans” configuration. Raman spectroscopy alone, however, is hardly capable of unambiguously identifying individual types of carotenoids occurring in biological samples [20], [26]. This difficulty mainly arises from the fact that carotenoids, in spite of the variety of terminal substituents characterising the individual carotenoid species, all share the same fundamental structure of conjugated C = C double bonds, which is responsible for the intense resonant Raman spectra. This holds true also for carotenoids present in *E. blumi*, for which no information other than the average number of conjugated C = C bonds and their conformation can be directly inferred from Raman data. The small intensity standard deviation for the Raman maps of *E. blumi*, however, yields information about the chemical homogeneity of carotenoid species present in this tardigrade species, suggesting the occurrence of carotenoids all having similar molecular structures. A more complete picture of the number and types of carotenoids present in *E. blumi* could perhaps be achieved by using several other wavelengths for Raman excitation (e.g. 413 nm and 488 nm) [27]. Although a multi-wavelength mapping analysis on the same animal could present some difficulties (the animal must be kept alive and still under the microscope for tens of minutes), this approach is definitely worth further investigation.

Since the Raman data presented clearly demonstrate the dietary origin of carotenoids in tardigrades, further information on the nature of the carotenoid pigments can be indirectly achieved from the analysis of the moss constituting the food of these animals. In particular, the identification of *Grimmia* moss as the source of carotenoids allows us to put forward specific hypotheses about the number and type of carotenoid species detected in the *E. blumi* specimens examined. Lutein, zeaxanthin, β-cryptoxanthin and β-carotene (Fig. S5 in SI) are the most commonly occurring carotenoids in mosses, and in particular they constitute up to ∼90% of the total carotenoids in *Grimmia* species [28]. In general, the position of the ν_1_ band in Raman spectra of carotenoids correlates well with the number of the conjugated double C = C bonds in their structure [20], [22], [23]; the average carotenoid spectra from *E. blumi*, and in particular spectra from the animal body cavity, well match the spectra of β-carotene, zeaxanthin and β-cryptoxanthin, all having 11 conjugated C = C bonds, but not that of lutein, which only has 10 [22], [27]. Moreover, previously reported chemical reactivity data of *Echiniscus* pigments [15] suggest that they do not include oxygen in their structure, leaving β-carotene as the most likely candidate for the pigmentation in the tardigrade of the genus *Echiniscus.*

Interestingly, the differences between the spectra of carotenoids localized in the gut and those in the body cavity are consistent with the presence of moss cellular material in the tardigrade gut. Such spectral differences could be explained considering a selective uptake of specific moss carotenoids by the tardigrade, as it has been reported for other animals [29], [30]. Indeed, Raman spectra from moss (both intact leaves or moss cellular material inside the tardigrade gut) must include spectral contributions from all its major carotenoids constituents, including lutein, whose ν_1_ band occurs at Raman shifts higher than 1521 cm^−1^ [22], [27]. Thus, the slight up-shift in the ν_1_ band position for those spectra collected from the gut with respect to spectra from the body cavity could be attributed to lutein, which is likely to be present in the moss cellular material inside the gut but might be less abundant or absent in the other organs of the tardigrade.

In general, the dietary origin of pigments in *E. blumi* also well explains the observed inter-individual variability in the overall carotenoid content, which may depend both on the quantity of food ingested and the time passed since the last food intake, assuming that carotenoids inside the animals are consumed. Since carotenoids are much less concentrated in the moss than in the *E. blumi* body cavity, this tardigrade appears to get these pigments from moss and accumulate them in its body cavity.

In animals, carotenoids have a diversity of functions: pigmentation with signalling purposes, immunity response, vision and protection against oxidative damage, among others [31]. In coloured tardigrades, the function of pigments is disputed.

Because they absorb UV and violet-blue light, carotenoids can act as light filters protecting against this harmful high-energy solar radiation [31], which is an indirect cause of oxidative damage to DNA, lipid membranes, and other cell components, through inducing the generation of free radicals [31]. The carotenoids present in tardigrades might well serve this photo-protective function. The observation that most tardigrade pigmented species dwell in sun-exposed habitats, such as the glacier surface [11] or on mosses and lichens growing on rocks located at high altitude and latitude, well support this hypothesis.

In *E. blumi*, the same carotenoids found in the body cavity are present in the eye-spots. Although ultrastructural data about these visual organs in tardigrades are available, the chemical nature of their pigments was previously unknown [12]. With the unique exception of a carotenoid photoactive protein found in cyanobacteria [32], carotenoids are not light receptors, but are nonetheless present in the eyes of several animals, as well as in humans, affording protection of photoreceptor cells against damaging radiation or contributing to obtain information regarding direction of the light [31]. Also the occurrence of carotenoids in the yolk of *E. blumi* eggs is not surprising, since these molecules are frequently found in high concentrations in eggs and in reproductive tissues of both vertebrates and invertebrates [31]. For example, the yolk of the eggs deposited above waterline by apple snails are supplied with adequate amount of carotenoids with photo-protective and antioxidant functions, to allow the development of embryos under harsh conditions such as desiccation and solar radiation exposure [33]. In both *E. blumi* eye-spots and eggs, carotenoids are likely to have photo-protective and antioxidant function as well. On the other hand, in view of the ultrastructural data available about tardigrades eye-spots and of the presence of the same carotenoids throughout the *E. blumi* body, we tend to exclude any role of these pigments as light receptors in this species.

Because of their well-known antioxidant functions, the carotenoids accumulated in the body cavity might help to protect the tardigrade from the dehydration-induced oxidative stress which occurs during the entering and remaining in anhydrobiosis [34], [35]. Organisms undergoing dehydration are known to suffer oxidative damage to their lipids, proteins and DNA, and tolerance to drying is correlated with an increase in their antioxidant potential [34], [36]. In *E. blumi*, the accumulation of free-radical scavengers such as carotenoids is likely to be one of the possible strategies, together with other mechanisms that include enzymes (e.g. peroxidases, catalases, superoxide dismutases) and antioxidants (e.g. glutathione) [36], [37] to fight the oxidative stress involved with anhydrobiosis. We simulated oxidative stress due to dehydration by H_2_O_2_ treatment, and then we tested if Raman micro-spectroscopy was able to measure variations in carotenoid content in living tardigrades. By measuring *in vivo* the radical quenching antioxidant action of carotenoids upon treatment with H_2_O_2_, Raman micro-spectroscopy shows how pigments are consumed in tardigrades exposed to oxidative stress. Such a method to monitor carotenoid variations could thus be used to further investigate *in vivo* the role of such molecules as antioxidants in tardigrades during real dehydration or other oxidative stress-related processes such as exposure to solar radiations.

### Conclusions

The method we propose, based on Raman imaging of living tardigrades, proves to be a reliable method for the investigation of the nature and function of pigments in tardigrades. It demonstrates that the pigments observed in the body (including eyes) and eggs of *E. blumi* are carotenoids (presumably β-carotene) which tardigrades obtain from the moss they feed on. The same spectroscopic method could be used to study other tardigrade species as well, extending our knowledge on the different kinds of pigments found in these animals in relation with their habitat and diet.

Moreover, we propose a role for carotenoids in tardigrades as scavengers for reactive oxygen species forming during exposition to solar radiations and/or dehydration processes. We tested this hypothesis *in vivo* by simulating the oxidative stress with a H_2_O_2_ treatment and measuring the decrease in carotenoid content. Considering the lack of methods to study directly the antioxidant function of carotenoids *in vivo* [31], pigmented tardigrades, investigated with Raman imaging, could be used as model organisms for this purpose.

## Supporting Information

Figure S1 **Average Raman spectra (black) together with the intensity standard deviation (grey) from a leaf of the moss G. orbicularis and from a tardigrade E. blumi.** The averages and standard deviation were calculated out of a set of 300 spectra for each sample, collected in the same experimental conditions (i.e. acquisition times, microscope objective, laser power).
(TIF)

Figure S2 **Percentage of survival of** ***E. blumi*** **specimens after 1 h and 24 h upon 15 min of exposure to different concentrations of hydrogen peroxide.** Bars correspond to standard deviation.
(TIF)

Figure S3 **Experimental group.** Histograms of the integrated Raman intensity in the 1460–1570 cm^−1^ region (i.e. integral of the most intense band) from Raman maps of 8 living *E. blumi* specimens before and after exposure to 25 mM of hydrogen peroxide for 15 min. For each histogram, the corresponding intensity Raman map depicting the carotenoid distribution (i.e. the intensity at 1521 cm^−1^) is shown as inset. White scale bars = 200 µm, color scale bars have units of counts. To the right, average spectra of Raman maps before (in red) and after (in blue) the treatment.
(TIF)

Figure S4 **Control group.** Histograms of the integrated Raman intensity in the 1460–1570 cm^−1^ region (i.e. integral of the most intense band) from Raman maps of 8 living *E. blumi* specimens before and after 15 min in water. For each histogram, the corresponding intensity Raman map depicting the carotenoid distribution (i.e. the intensity at 1521 cm^−1^) is shown as inset. White scale bars = 200 µm, color scale bars have units of counts. To the right, average spectra of Raman maps before (in red) the treatment and after (in blue) the treatment.
(TIF)

Figure S5 **Chemical structures of the main carotenoids (up to ∼90% of the total carotenoids) found in** ***Grimmia*** **mosses (Czeczuga B, 1980,** ***The Bryologist*** **83∶21–28).**
(TIF)

Figure S6 **Total integrated Raman intensity in the 1460–1570 cm^−1^ region (summed over the whole map) as an indicator of the total carotenoids content before (black bars) and after (white bars) exposure to hydrogen peroxide solution (A, experimental group) or water (B, control group) for a total of 16** ***E. blumi*** **specimens.**
(TIF)
