# Supplementary figures and images for: Nature, Source and Function of Pigments in Tardigrades: In Vivo Raman Imaging of Carotenoids in Echiniscus blumi
Source: PLoS One. 2012 Nov 21;7(11):e50162. doi: 10.1371/journal.pone.0050162 (PMC3503987; doi:10.1371/journal.pone.0050162)

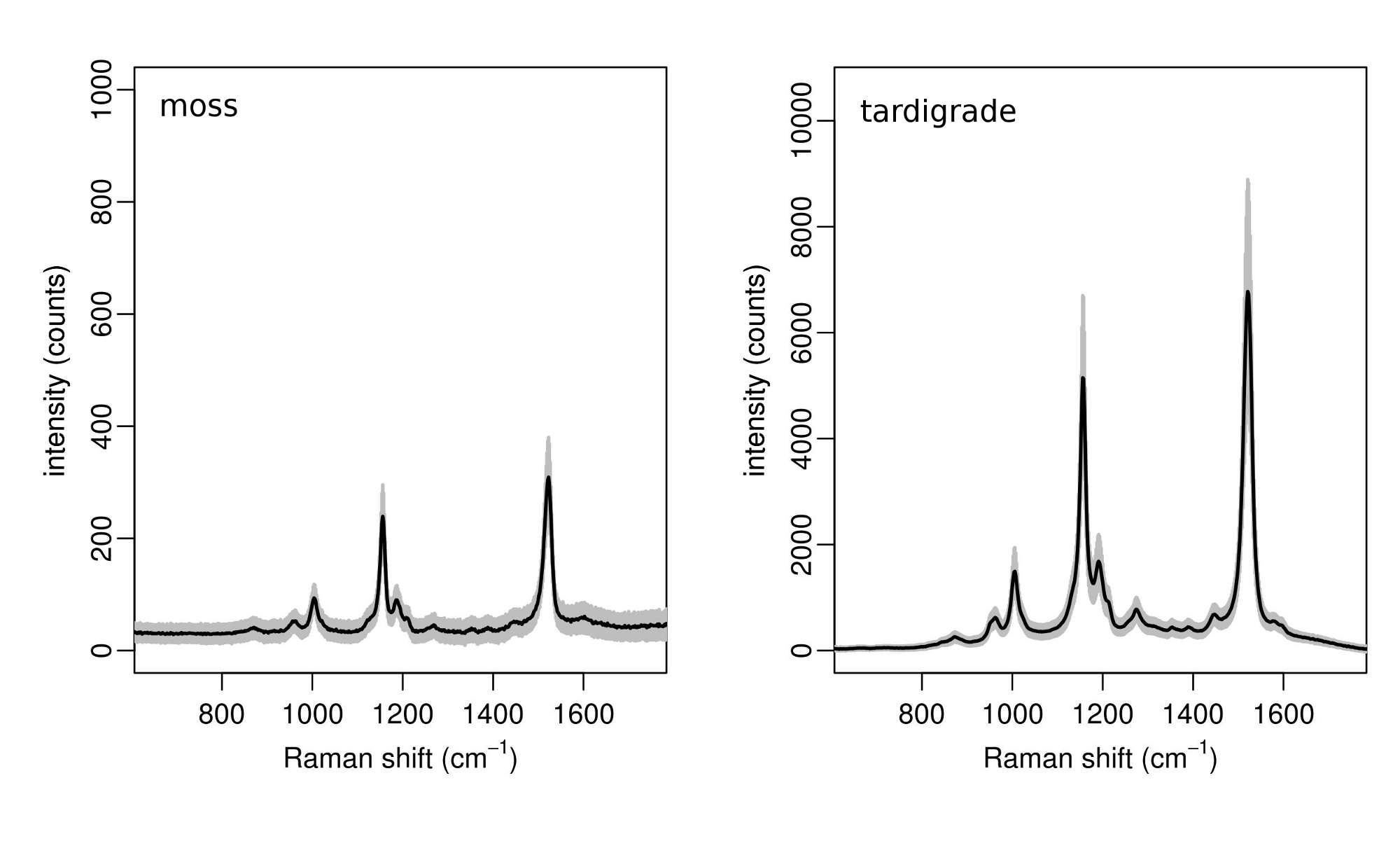

Supplement: Figure S1 — Average Raman spectra (black) together with the intensity standard deviation (grey) from a leaf of the moss G. orbicularis and from a tardigrade E. blumi. The averages and standard deviation were calculated out of a set of 300 spectra for each sample, collected in the same experimental conditions (i.e. acquisition times, microscope objective, laser power). (TIF) [file pone.0050162.s001.tif]

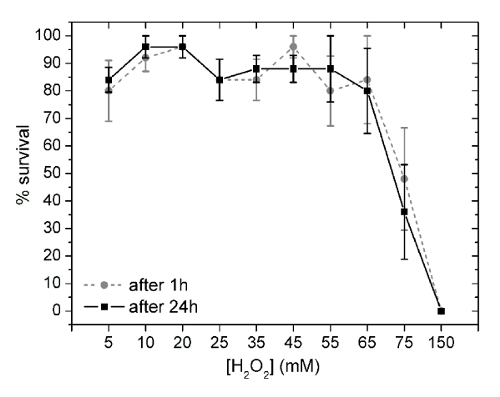

Supplement: Figure S2 — Percentage of survival of E. blumi specimens after 1 h and 24 h upon 15 min of exposure to different concentrations of hydrogen peroxide. Bars correspond to standard deviation. (TIF) [file pone.0050162.s002.tif]

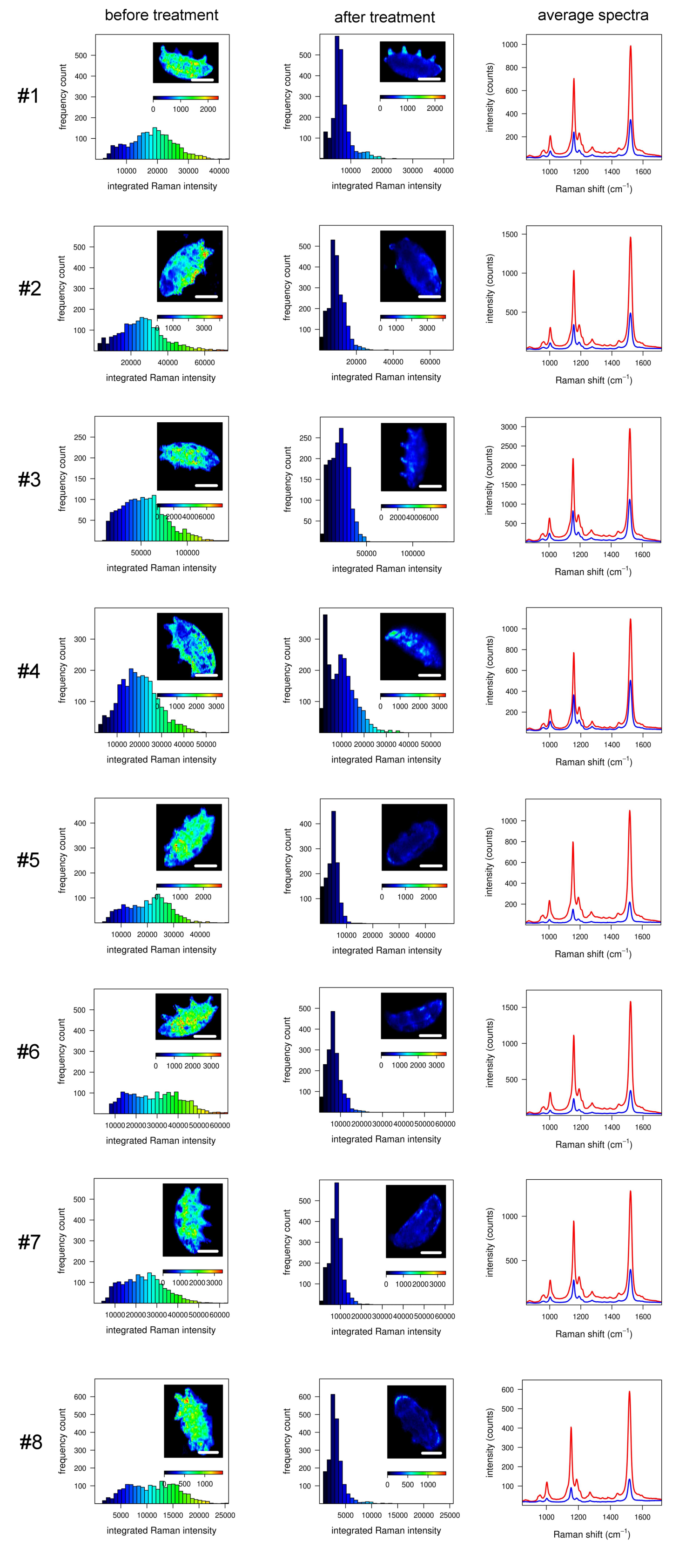

Supplement: Figure S3 — Experimental group. Histograms of the integrated Raman intensity in the 1460–1570 cm−1 region (i.e. integral of the most intense band) from Raman maps of 8 living E. blumi specimens before and after exposure to 25 mM of hydrogen peroxide for 15 min. For each histogram, the corresponding intensity Raman map depicting the carotenoid distribution (i.e. the intensity at 1521 cm−1) is shown as inset. White scale bars = 200 µm, color scale bars have units of counts. To the right, average spectra of Raman maps before (in red) and after (in blue) the treatment. (TIF) [file pone.0050162.s003.tif]

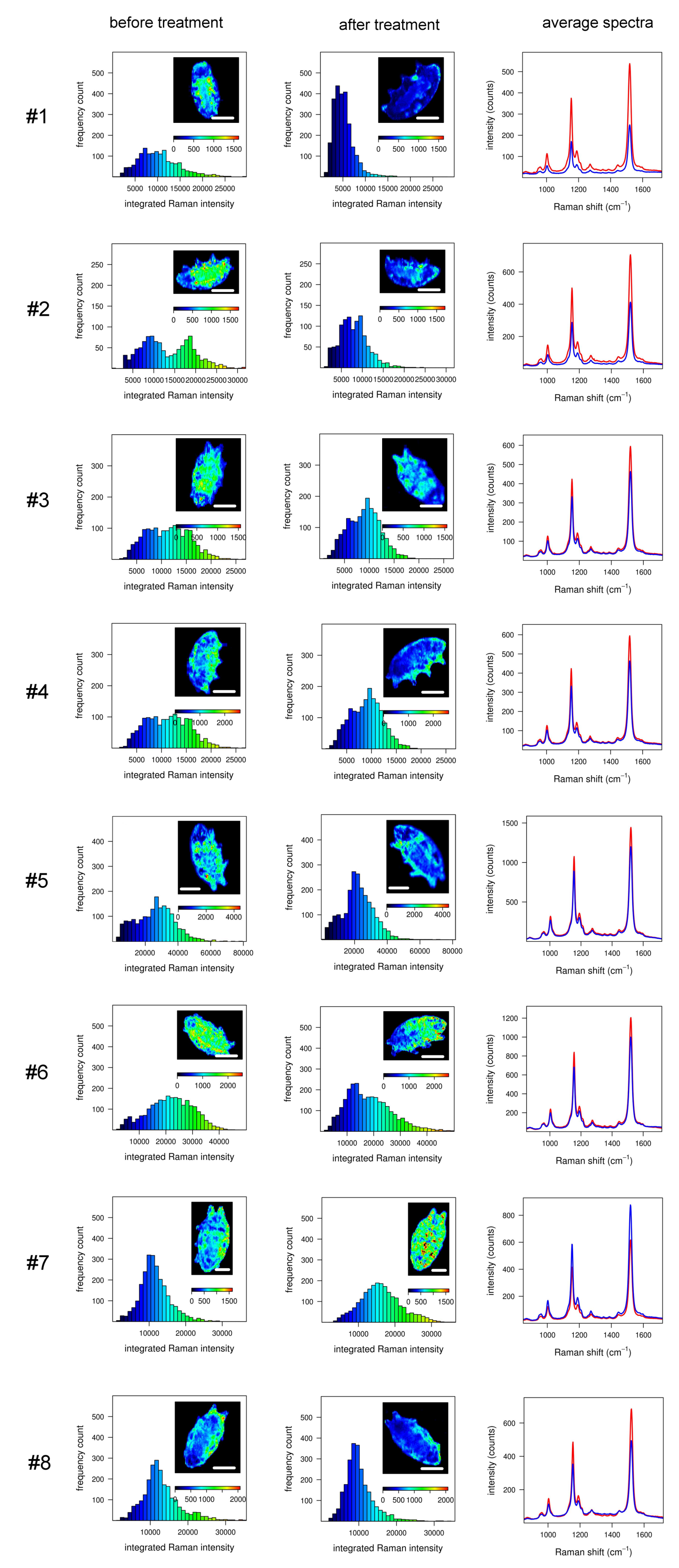

Supplement: Figure S4 — Control group. Histograms of the integrated Raman intensity in the 1460–1570 cm−1 region (i.e. integral of the most intense band) from Raman maps of 8 living E. blumi specimens before and after 15 min in water. For each histogram, the corresponding intensity Raman map depicting the carotenoid distribution (i.e. the intensity at 1521 cm−1) is shown as inset. White scale bars = 200 µm, color scale bars have units of counts. To the right, average spectra of Raman maps before (in red) the treatment and after (in blue) the treatment. (TIF) [file pone.0050162.s004.tif]

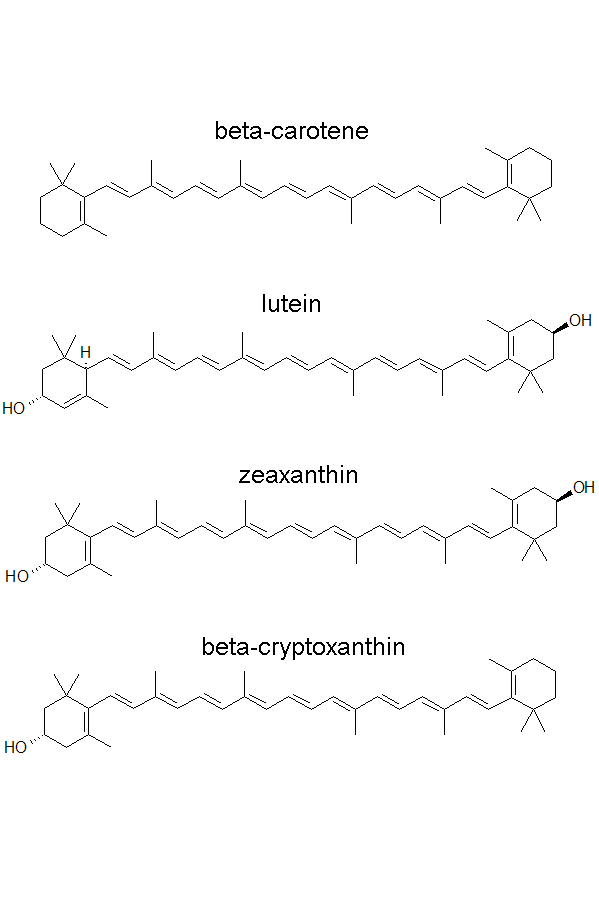

Supplement: Figure S5 — Chemical structures of the main carotenoids (up to ∼90% of the total carotenoids) found in Grimmia mosses (Czeczuga B, 1980, The Bryologist 83∶21–28). (TIF) [file pone.0050162.s005.tif]

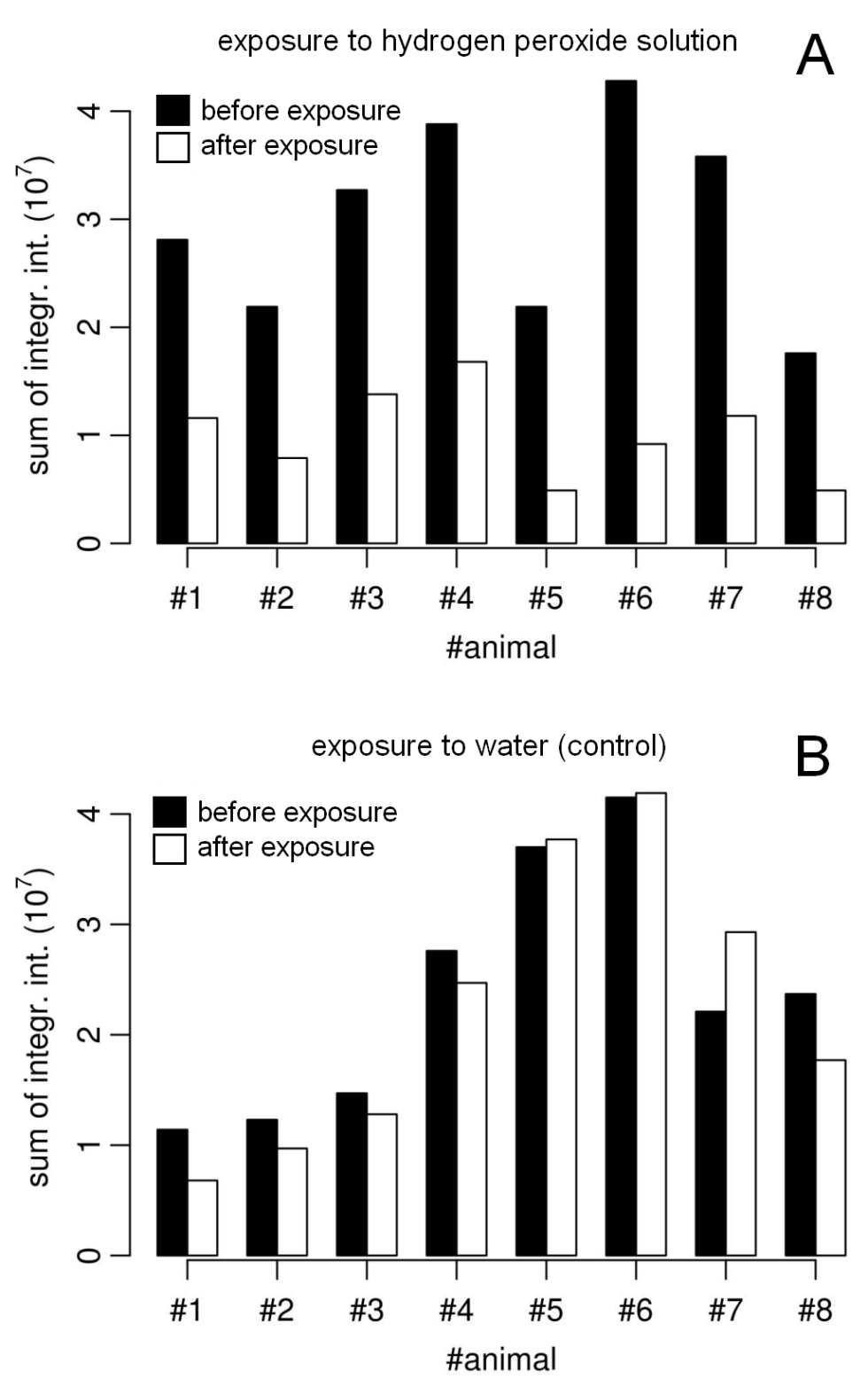

Supplement: Figure S6 — Total integrated Raman intensity in the 1460–1570 cm−1 region (summed over the whole map) as an indicator of the total carotenoids content before (black bars) and after (white bars) exposure to hydrogen peroxide solution (A, experimental group) or water (B, control group) for a total of 16 E. blumi specimens. (TIF) [file pone.0050162.s006.tif]
